# Effect of RGO-Y_2_O_3_ and RGO-Y_2_O_3_:Cr^3+^ nanocomposite sensor for dopamine

**DOI:** 10.1038/s41598-021-87749-z

**Published:** 2021-04-30

**Authors:** J. K. Shashikumara, Bhimanagouda Kalaburgi, B. E. Kumara Swamy, H. Nagabhushana, S. C. Sharma, P. Lalitha

**Affiliations:** 1 Department of P.G. Studies and Research in Industrial Chemistry, Kuvempu University, Jnana Sahyadri, Shankaraghatta, Shimoga, Karnataka 577451 India; 2 Department of Studies and Research in Physics, Tumkur University, Tumkur, Karnataka 572 103 India; 3 National Assessment and Accreditation Council (Work Carried Out as Honorary Professor), Jain University, Bangalore, Karnataka 560 069 India; 4 Distinguished Professor in the Centre for Energy, Indian Institute of Technology Guwahati, Guwahati, India; 5 Department of Chemistry, Avinashilingam Institute for Home Science and Higher Education for Women, Coimbatore, Tamil Nadu 641043 India

**Keywords:** Materials science, Techniques and instrumentation, Characterization and analytical techniques

## Abstract

The RGO-Y_2_O_3_ and RGO-Y_2_O_3_: Cr^3+^ (5 mol %) nanocomposite (NC) synthesized by hydrothermal technique. The structure and morphology of the synthesized NCs were characterized by X-ray diffraction (XRD), scanning electron microscopy (SEM), and transmission electron microscopy (TEM). Y_2_O_3_:Cr^3+^ displays spherical-shaped particles. Conversely, the surface of the RGO displays a wrinkly texture connecting with the existence of flexible and ultrathin graphene sheets. The photoluminescence (PL) emission spectra showed series of sharp peaks at 490, 591, and 687 nm which corresponding to ^4^F_9/2_ → ^6^H_15/2_, ^4^F_9/2_ → ^6^H_13/2,_ and ^4^F_9/2_ → ^6^H_11/2_ transitions and lies in the blue, orange, and red region. The prepared NCs were used for the preparation of modified carbon paste electrodes (MCPE) in the electrochemical detection of dopamine (DA) at pH 7.4. Both modified electrodes provide a good current response towards voltammetric detection of DA. Doping is an effective method to improve the conductivity of Y_2_O_3_:Cr^3+^ and developed a method for the sensor used in analytical applications.

## Introduction

Nanotechnology has become one of the most important areas in science. The nanoparticles (NPs) exhibit unique chemical, physical, and electronic properties that are different from those of bulk materials, due to their small size and better architecture. Electrochemical detection is highly attractive for the monitoring of biomolecules and infectious diseases^1–4^.

Green chemistry controlled and bio-compatible way offers savvy, eco-friendly and scaled up for large quantity and do not need extreme conditions such as high temperature, pressure, and hazardous chemicals to fabricate NPs of metal, metal oxide as well as carbon comprising graphene^5^.

As of late, Graphene is one of the developing materials with a single layer of carbon in a closed pressed honeycomb two-dimensional lattice materials having one of kind properties such as large surface area, improved electrical conductivity, unrivaled electrochemical property, and chemically stability make the graphene as profoundly encouraging for a wide range of applications, namely, energy storage, sensor, drug delivery, and optoelectronic devices, etc.^6–10^.

Nonetheless, a vital task in the fabrication and treating of graphene is that irreversible agglomerates and or restack to frame graphite as a result of van der-Waals force interaction. Graphene oxide (GO) is a major by-product of graphene. The exterior of the GO sheets has a huge quantity of oxygen functional groups namely hydroxyl, carboxyl and epoxy groups situated at the edge of the sheets makes the GO sheet powerfully hydrophilic, permitting them to freely scatter in water^11^.

Furthermore, the GO sheet is considered to be an electrical shielding material owing to its disordered sp^2^ bonding network and can be reestablished by attainment the reduction of GO. In recent advances, reduced graphene oxide (RGO) is used as the auxiliary for pristine graphene since of its large surface area and superior conductivity, which has been in favor of the precise revealing of the biological analytes. Additionally, RGO also comprises various types of oxygen vacancies, which make it possible to be altered with other NMs to additional progress its chemical as well as physical properties for the future generation point-of-care biosensors (POC) as well as energy storage devices. Reduction of GO results in the partial restoration of graphitic network conventionally attained using chemical, thermal, and electrochemical pathways^12^.

Generally, for chemical reduction of GO, reducing agents (RA) such as hydrazine (N_2_H_4_), hydrazine hydrate, and sodium borohydride (NaBH_4_) were used. Conversely, these RA are unsafe to human health and the environment. Further, topological defects and vacancies were created upon thermal reduction of the GO sheet. The presence of these defects which affects the electronic properties of the RGO, resulting in the decrease of ballistic transport path length and introduce scattering sites. Therefore, the execution of a greener reduction route can offer a viable substitute methodology for large production of RGO^13^. Other than the utilization of GO and RGO, these days a lot of consideration for the integration of inorganic phosphor with GO to manufacture composites or hybrids has become a hotly debated issue of exploration because of their upgraded functionalities that cannot be accomplished by either part alone. It is well known that the attachment of inorganic NPs onto GO may inhibit the aggregation and improve the significant persuasive effect on electrochemical properties through attaching them onto GO sheets. Amongst the metal oxide NPs decorating RGO, much attention has been given to doped and undoped NPs especially the stable Y_2_O_3_ host^14,15^.

Till date, many approaches have been utilized for the fabrication of MO/RGO composites namely microwave, hydrothermal, pyrolysis method, etc.^16–20^ when the addition of MO NPs to the GO matrix, an upsurge in porosity happens and the GO-MO attains the properties that are dissimilar from those exhibited by each distinct component. On the other hand, Y_2_O_3_ NPs are chemically steady and have a narrow bandgap that enables electron transfer and offers excellent electrochemical sensitivity.

The present work RGO-Y_2_O_3_ and RGO-Y_2_O_3_: Cr^3+^ NCs synthesized by hydrothermal technique. The prepared NCs were well characterized by X-ray diffraction (XRD), scanning electron microscopy (SEM), and transmission electron microscopy (TEM). NCs were used as the electrochemical sensor for voltametric determination of DA. The developed sensors show good current sensitivity towards DA. The prepared RGO-Y_2_O_3_ and RGO-Y_2_O_3_: Cr^+3^ MCPEs provide good selectivity, high sensitivity, and excellent stability and reproducibility over several days. In addition to comparably doping is an effective method to improve the conductivity of Y_2_O_3_:Cr^3^^+^ and doping shows good linearity, sensitivity, lower detection limit compare to undoped NCs towards DA. Finally, the modified electrodes were utilized for the detection DA in biological samples.

## Experimental section

### Reagents & investigation techniques

The sodium hydroxide, Dopamine hydrochloride, Uric acid, Na_2_HPO_4_ & NaH_2_PO_4_ was from nice chemicals. The graphite powder (Loba Chemie), silicon oil (Himedia) & all other required solutions were prepared from distilled water. The electrochemical experiments were performed on a voltammetric instrument of model CHI-660c (CH Instrument-660 electrochemical workstation). The Shimadzu-made diffractometer provided with CuKα radiation was utilized for structural characterization. Surface morphology and particle size were studied with the help of a field emission scanning electron microscope (FESEM, TESCON) and transmission electron microscope (TEM, H-600, Hitachi, Japan) respectively. The Horiba made spectrofluorimeter (Jobin Yvon) was used for Photoluminescence (PL) with a 450 W Xenon lamp as an excitation source.

### Preparation of RGO-Y_2_O_3_ composite using hydrothermal synthesis

The chemicals procured in the present work are analytical grade and used without further purification. The modified Hummers method has been used for the synthesis of GO^21^. The obtained GO solution was sonicated for about 25–30 min, centrifuged to wipe off the unreacted GO.

A single-step hydrothermal route was utilized for the fabrication of pure Y_2_O_3_ and Cr doped Y_2_O_3_ in RGO. In a typical synthesis, stoichiometric quantities of yttrium chloride (YCl_3_·6H_2_O) and chromium chloride (CrCl_3_·6H_2_O) were dissolved to 120 mL of mixed liquid, encompassing 60 mL of C_2_H_5_OH and 60 mL of GO aqueous dispersion before stirred for 25–30 min to get a clear solution. Then the resulting solution was shifted to a 180 mL Teflon-lined stainless-steel autoclave and treated thermally at 175 °C for 12 h. Afterward, the autoclave was naturally cooling down to room temperature. The final yield was washed away several times with distilled water and then ethanol, separately, and then centrifugation. The as-obtained products were dried at 80 °C for 12 h in air, and annealed at 500 °C for 2 h in Ar and subsequently 200 °C for 12 h in the air to form the RGO/Y_2_O_3_:Cr^3+^ NCs. Pure Y_2_O_3_ and a series of RGO-Y_2_O_3_:Cr^3+^ NCs with the GO contents of 2, 4, 6, 8, and 10 mg were synthesized by changing the concentration of GO aqueous dispersion.

### Preparation of BCPE and MCPEs

**Scheme 1:**
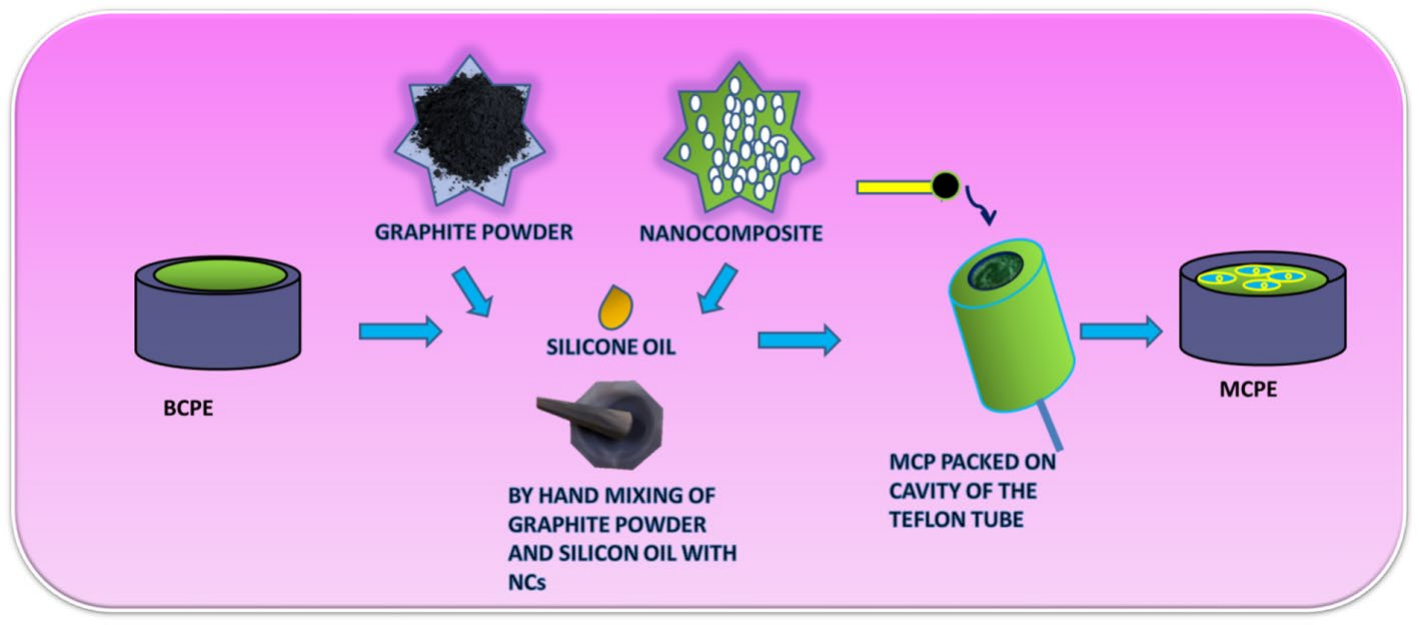
Schematic representation of the stepwise fabrication of electrode.

The BCPE was prepared by hand mixing of graphite powder and silicon oil at the ratio of 70:30 (w/w) in an agate mortar for about 30 min until a homogenous paste was obtained. The prepared carbon paste was tightly packed into a PVC tube (3 mm internal diameter) and the electrical contact was provided by a copper wire connected to the paste at the end of the tube and polished using smooth paper^22,23^. The CPE was modified by taking different weights of RGO-Y_2_O_3_ and RGO-Y_2_O_3_Cr^3+^ NCs (2, 4, 6, 8, and 10 mg) in silicon oil and graphite powder. Then this mixture was thoroughly mixed in an agate mortar for about 30 min and packed into a homemade Teflon cavity current collector and polished using soft paper (Scheme 1)^24^.

## Results and discussion

### Characterization of RGO-Y_2_O_3_ and RGO-Y_2_O_3_Cr^3+^ NCs

**Figure 1:**
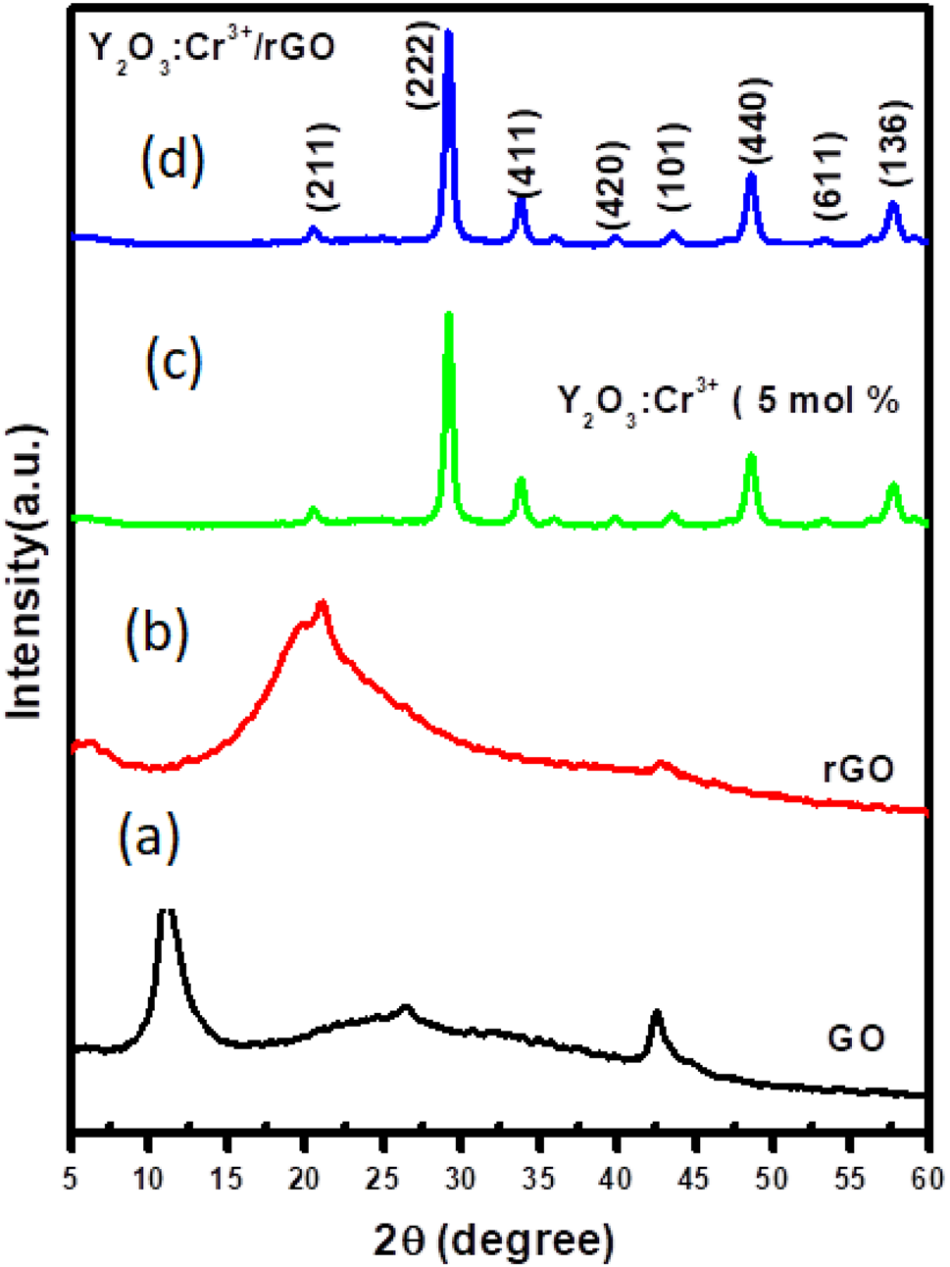
PXRD patterns of (**a**) GO (**b**) rGO NCs (**c**) Y_2_O_3_:Cr^3+^ NCs(**d)** rGO-Y_2_O_3_:Cr^3+^ NCs.

Figure 1 shows the powder X-ray diffraction (PXRD) patterns of GO, rGO, Y_2_O_3_:Cr^3+^ and RGO/Y_2_O_3_:Cr^3+^ NCs. The GO exhibited a distinctive (001) peak at 2θ = 11° (JCPDS No. 89-8490) with an interlayer distance of 0.8 nm, which is larger than the interlayer distance of graphite (0.34 nm), revealing that many different oxygen-containing groups were intercalated within the interlayer space. The peak at 11° completely disappeared after annealing, replaced by a broad peak at 2θ = 22° for rGO (JCPDS No. 89-7213), with a d-spacing of 0.4 nm, implying that the successful reduction of GO to reduced graphene oxide. Further, Y_2_O_3_:Cr^3+^/RGO NCs (Fig. 1d) were dominated by the Y_2_O_3_:Cr^3+^ reflections (JCPDS file no. 83-0134). Most of the PXRD peaks of the Y_2_O_3_:Cr^3+^/RGO hybrids were similar to those of the Y_2_O_3_:Cr^3+^ NPs with minor variations in the peak positions (broadening, change in the intensity of the diffraction profiles, which showed that Y_2_O_3_:Cr^3+^ is effectively anchored onto RGO which was auxiliary proved by the absence of a sharp (002) diffraction peak in the Y_2_O_3_:Cr^3+^/RGO hybrid. Hence, the restacks of RGO are inhibited by the control of Y_2_O_3_:Cr^3+^ NPs.

**Figure 2:**
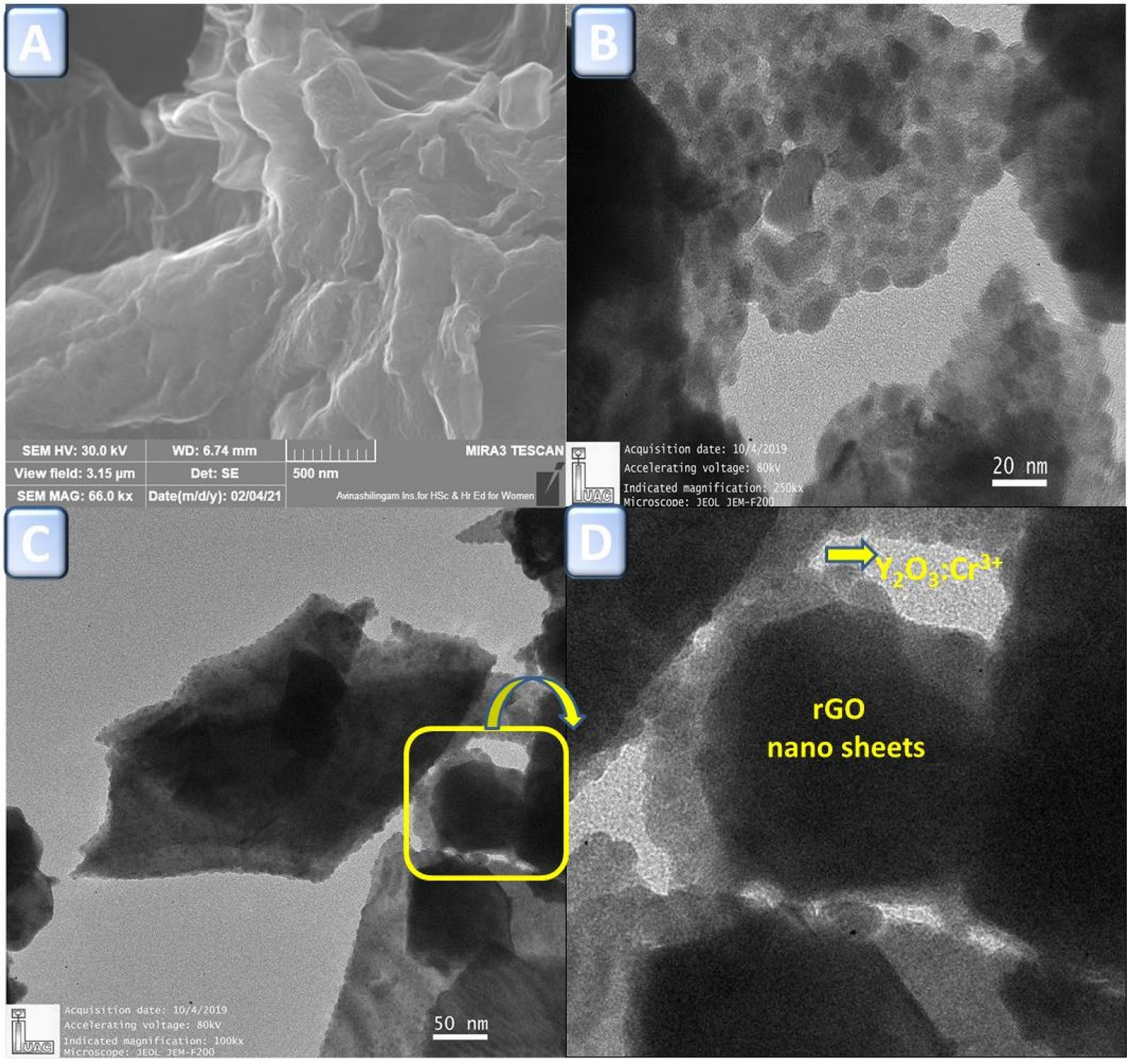
**(A**) FESEM image of rGO nanosheet, (**B**) TEM image of Y_2_O_3_:Cr^3+^ NPs, (**C**) TEM image of rGO/Y_2_O_3_:Cr^3+^ NCs**. (D**) Enlarged image of rGO/Y_2_O_3_:Cr^3+^ NCs.

Figure 2a shows the typical FESEM image of the rGO. As can be seen from the figure, the ultrathin, crumpled nanosheets are found to be transparent and wrinkled like wavy silk veils with randomly arranged and overlapped with each other. Figure 2b,c shows the TEM images of Y_2_O_3_:Cr^3+^ nanopowders and rGO/Y_2_O_3_:Cr^3+^ NCs respectively. It is clearly evident that the nanopowders exhibit almost spherical shaped particles. However, in the case of NCs, the spherical shaped particles are uniformly distributed in rGO sheets. The inset image (Fig. 2d) confirms the formation of rGO/Y_2_O_3_:Cr^3+^ NCs and RGO nanosheets are well interposed with Y_2_O_3_:Cr^3+^ NPs.

### Photoluminescence analysis

**Figure 3:**
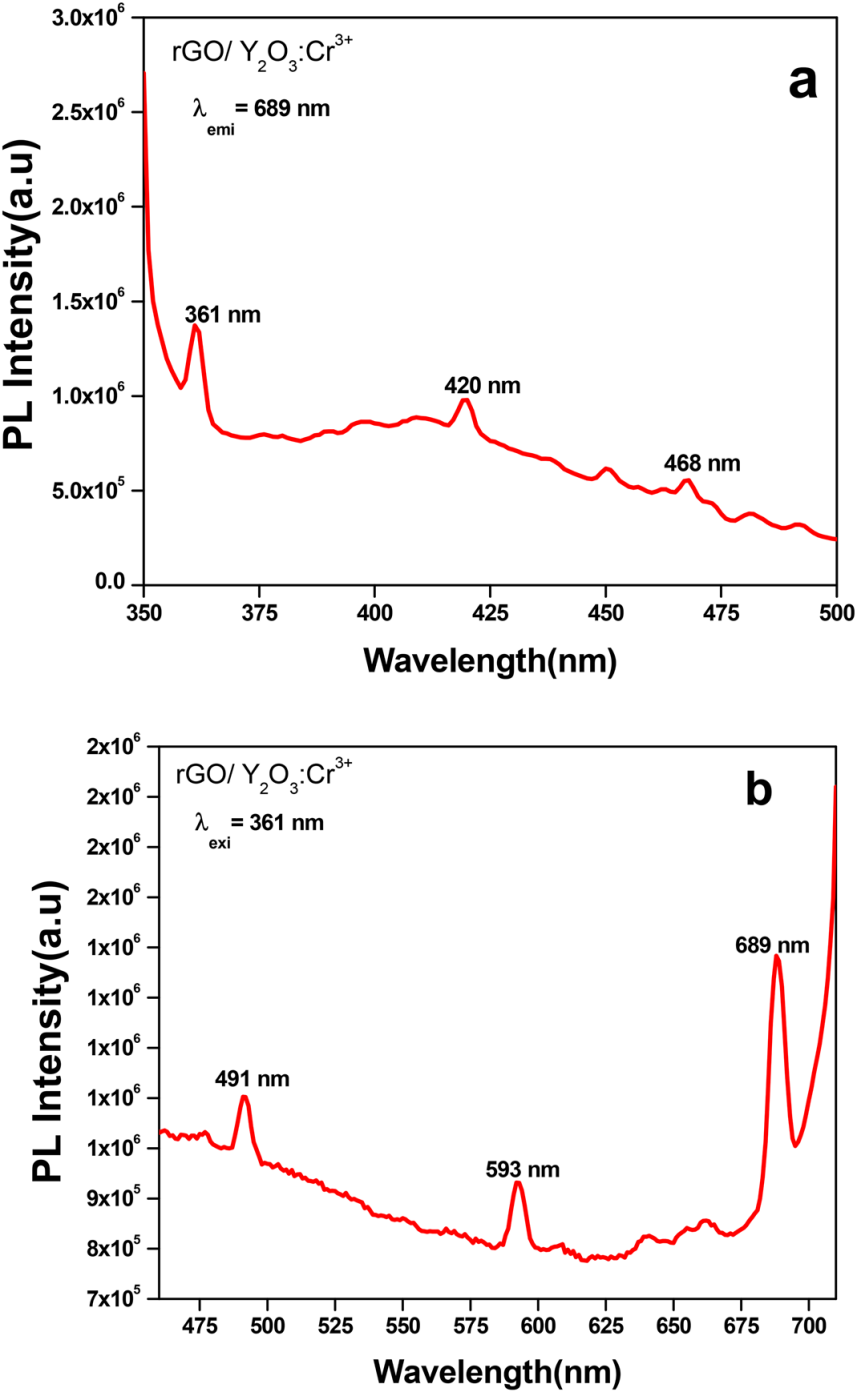
**(a)** PL excitation spectrum, (**b)** emission spectrum of rGO/Y_2_O_3_:Cr^3+^ NCs.

Figure 3a shows the excitation spectrum of Y_2_O_3_: Cr^3+^ (5 mol%) NPs monitored at 689 nm emission wavelength. The excitation spectrum consists of peaks at 361 nm 419 nm corresponding to the transitions ^6^H_15/2_ → ^4^P_7/2_ and ^6^H_15/2_ → ^4^M_21/2_ respectively. Typical PL emission spectra of Y_2_O_3_: Cr^3+^ (5 mol%) NPs under 361 nm excitation wavelength are shown in Fig. 3b. The emission spectra displays sharp peaks at ~ 490, 591, and 689 nm corresponding to ^4^F_9/2_ → ^6^H_15/2_, ^4^F_9/2_ → ^6^H_13/2,_ and ^4^F_9/2_ → ^6^H_11/2_ transitions which lie in the blue, orange and red region. As can be seen from the figure, it is apparent that the red emission was dominating when compared to blue and orange emissions. The transition corresponding to orange emission is magnetically allowed and hardly differs with the crystal field strength around the Cr^3+^ ion. Whereas, the transition corresponding to blue emission belongs to the hypersensitive electric field (forced electric dipole) transition with the selection rule *ΔJ* = 2, which is strongly influenced by the outside surrounding environment. When Cr^3+^ is located at a low—symmetry local site (without inversion center), red emission is often dominant. In the present study, since the red emission is dominant, the Cr^3+^ ions occupy lower symmetry local site in the Y_2_O_3_ host matrix.

### Characterization and optimization of RGO-Y_2_O_3_ and RGO-Y_2_O_3_: Cr^3+^ MCPE

**Figure 4:**
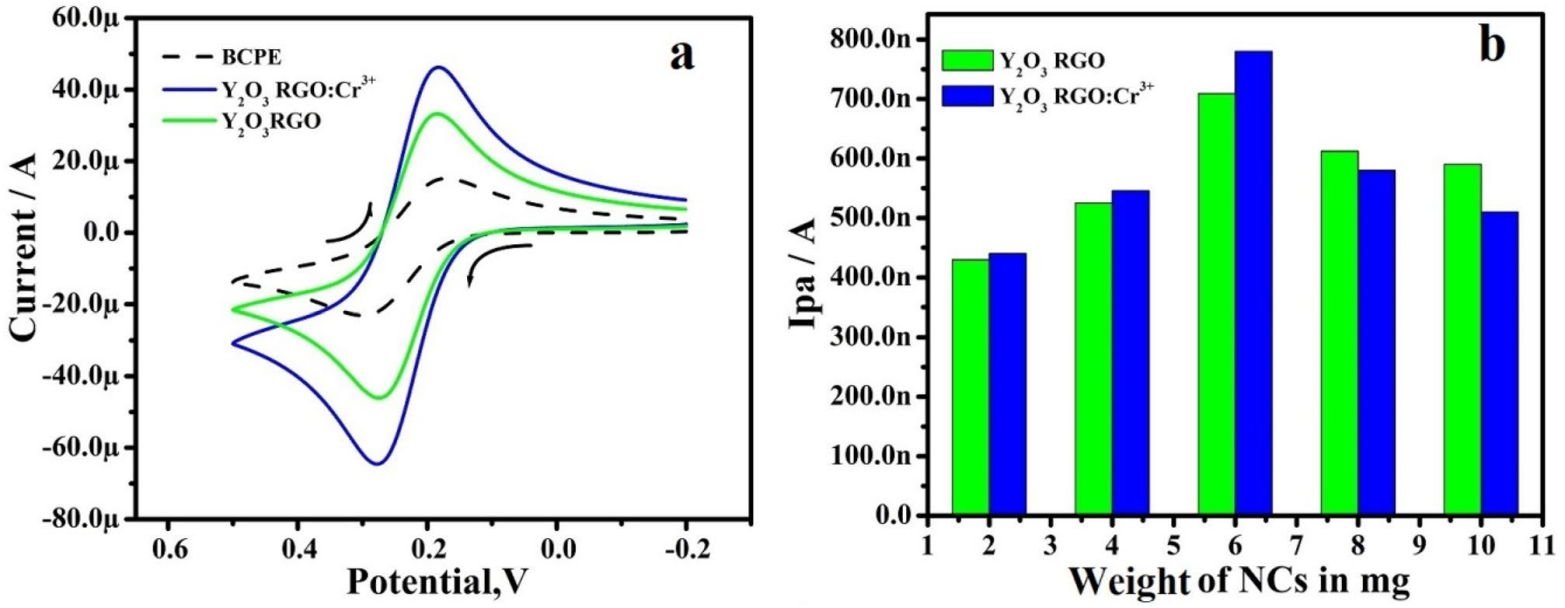
** (a)** CVs of 1 mM potassium ferrocyanide in 1 M KCl solution at scan rate 50 mVs^-1^. (**b)** Plot of Ipa versus weight of NCs.

Figure 4a establishes the CVs response of 1 mM K_4_ [Fe (CN)_6_] in the KCl at the modified electrodes. The NCs MCPE displayed increment in the anodic peak current (Ipa) with the decrease ΔEp compared to BCPE and RGO-Y_2_O_3_: Cr^3+^ MCPE exhibit highest Ipa. The total active surface area of electrodes calculated by Randles-Sevick’s equation (1)^25^. The area calculated that of greater value for NCs MCPE (0.0412 cm^2^ and 0.0456 cm^2^) compared to BCPE (0.031 cm^2^).

1 $${\text{I}}_{{\text{p}}} = \, \left( {{2}.{69} \times { 10}^{{5}} } \right){\text{ n}}^{{{3}/{2}}} {\text{A D}}_{0}^{{{1}/{2}}} {\text{C}}_{0} {\upsilon}^{{{1}/{2}}}$$

To optimize the quantity of RGO-Y_2_O_3_ and RGO-Y_2_O_3_Cr^+3^ were used as a modifier, CPEs modified with different quantities of the RGO-Y_2_O_3_ and RGO-Y_2_O_3_ composites were used to determine 10 µM of DA (PBS at pH 7.4,). Figure 4b depicts the plot of Ipa versus the different quantities of RGO-Y_2_O_3_ and RGO-Y_2_O_3_Cr^3+^ NPs respectively. As a result, the MCPEs modified with 6 mg were used as optimized electrodes for further electrochemical investigations.

### The electrochemical response of DA at RGO-Y_2_O_3_ and RGO-Y_2_O_3_: Cr^3+^ MCPE

**Figure 5:**
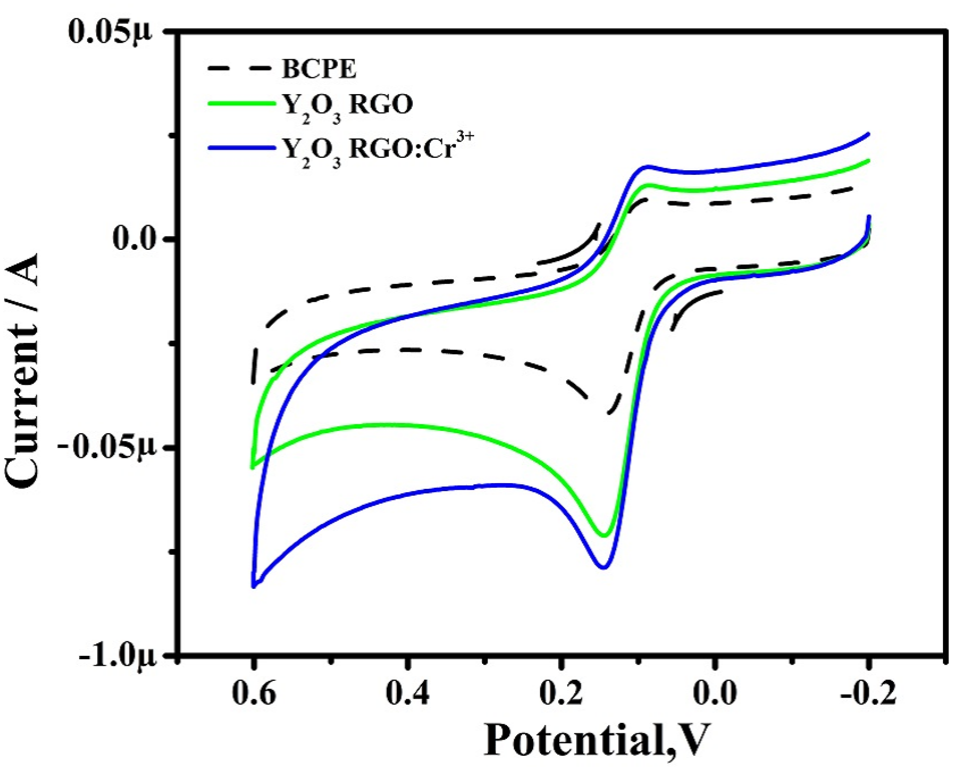
CVs of 10 μM DA in 0.2 M PBS solution of pH 7.4 at scan rate 50 mVs^-1^.

Figure 5 shows the current response of 10 µM DA at the BCPE and RGO-Y_2_O_3_ and RGO-Y_2_O_3_: Cr^3+^ MCPEs (0.2 M PBS at pH 7.4) at SR of 50 mVs^-1^. In BCPE, the CVs of DA showed a minimum current response, and a peak potential was noted at 142 mV and 86 mV, respectively and their corresponding redox peak potential differences (ΔEp) were computed to be 56 mV. Similarly, RGO-Y_2_O_3_ and RGO-Y_2_O_3_:Cr^3+^ MCPE showed good Ipa with decreased ΔEp value 54 mV and 52 mV respectively as compared to the BCPE. It exhibits clear proof of the catalytic effect of the expected sensor towards DA investigation.

### Response of scan rate at RGO-Y_2_O_3_ and RGO-Y_2_O_3_:Cr^3+^ MCPE

**Figure 6:**
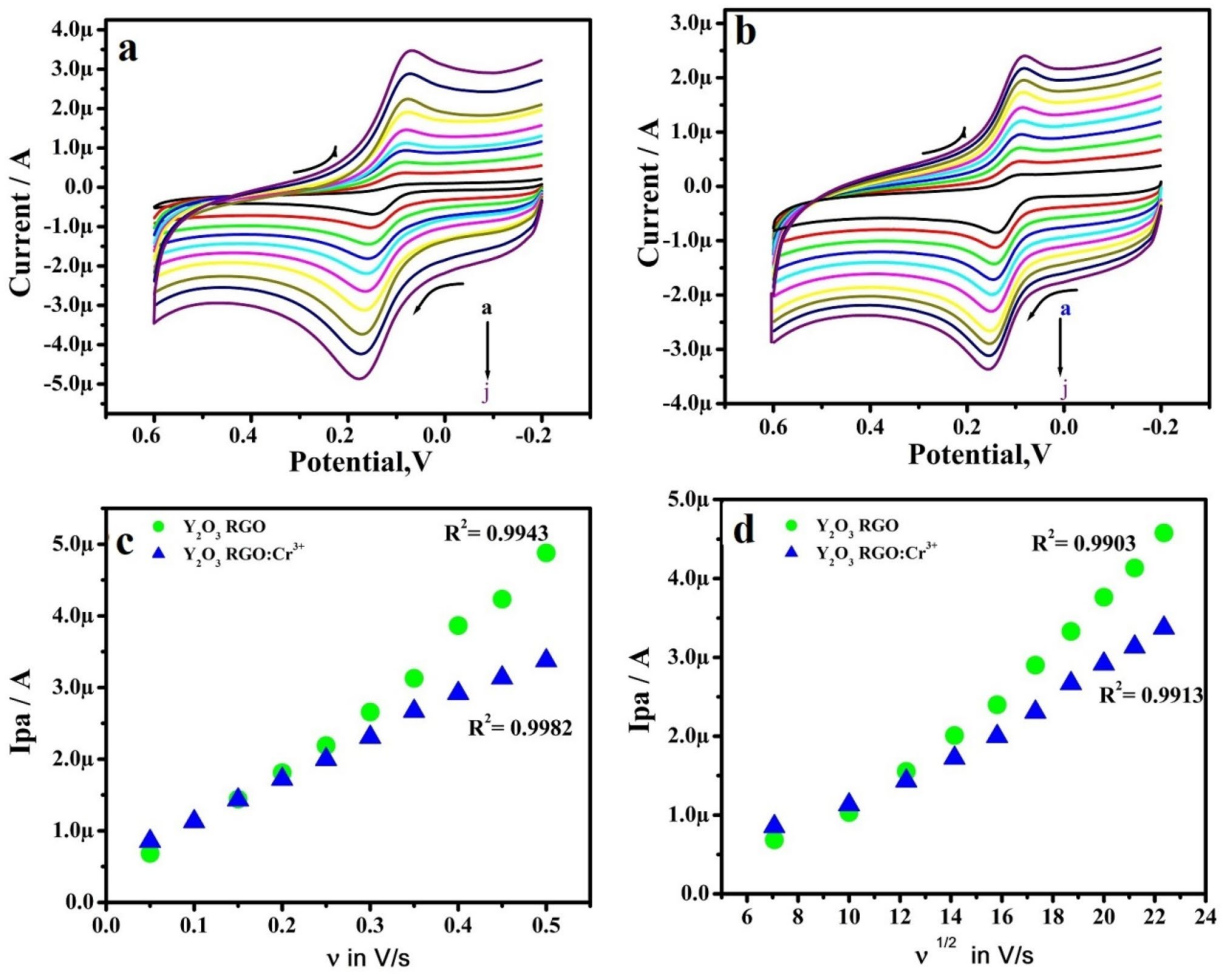
**(a)** CVs of 10 μM DA (0.2 M PBS of pH 7.4) at RGO-Y_2_O_3_ MCPE at various scan rate (**b)** CVs of 10 μM DA (0.2 M PBS of pH 7.4) at RGO-Y_2_O_3_; Cr^3+^ MCPE at various scan rate. (**c)** Plot of Ipa versus υ. (**d)** Plot of Ipa versus υ^1/2^.

The scan rate (υ) was inspected by CV using 10 μM DA in 0.2 M PBS at RGO-Y_2_O_3_ and RGO-Y_2_O_3_; Cr^3+^ MCPE. Figure 6a &b shows the Ip increment with a slight positive shift in the peak potential when the υ was a hike in the range from 50 to 500 mVs^-1^. The kinetics of the electrode was evaluated by plotting of υ v/s Ipa presents marvelous linearity (Fig. 6c). The υ^1/2^ v/s Ipa (Fig. 6d) also shows good linearity. This suggests the adsorption-controlled phenomena on electrodes^26^. The heterogeneous rate constant (k^0^) (Table 1) of RGO-Y_2_O_3_ and RGO-Y_2_O_3_Cr^+3^ MCPEs was calculated by using equation (2)^26^.

2 $$\Delta {\rm{Ep}} = 201.39 {\rm log (\upsilon/k}^{0} ) - 301.78$$

**Table 1:** The rate constant values for DA at different sweep rates.

υ (mV/s)	ΔEp (mV)		k°(s^−1^)	
	RGO-Y_2_O_3_	RGO-Y2O_3_;Cr^3+^	RGO-Y_2_O_3_	RGO- Y2O_3_;Cr^3+^
50	74	53	0.68083	0.86559
100	74	53	1.36166	1.73119
150	76	54	1.99631	2.56726
200	78	60	2.60157	3.19606
250	82	63	3.10659	3.86037
300	86	65	3.56126	4.52771
350	90	71	3.96907	4.93211
400	97	71	4.18718	5.6367
450	102	74	4.44884	6.12746
500	108	75	4.61542	6.73089

### Success of concentration

**Figure 7:**
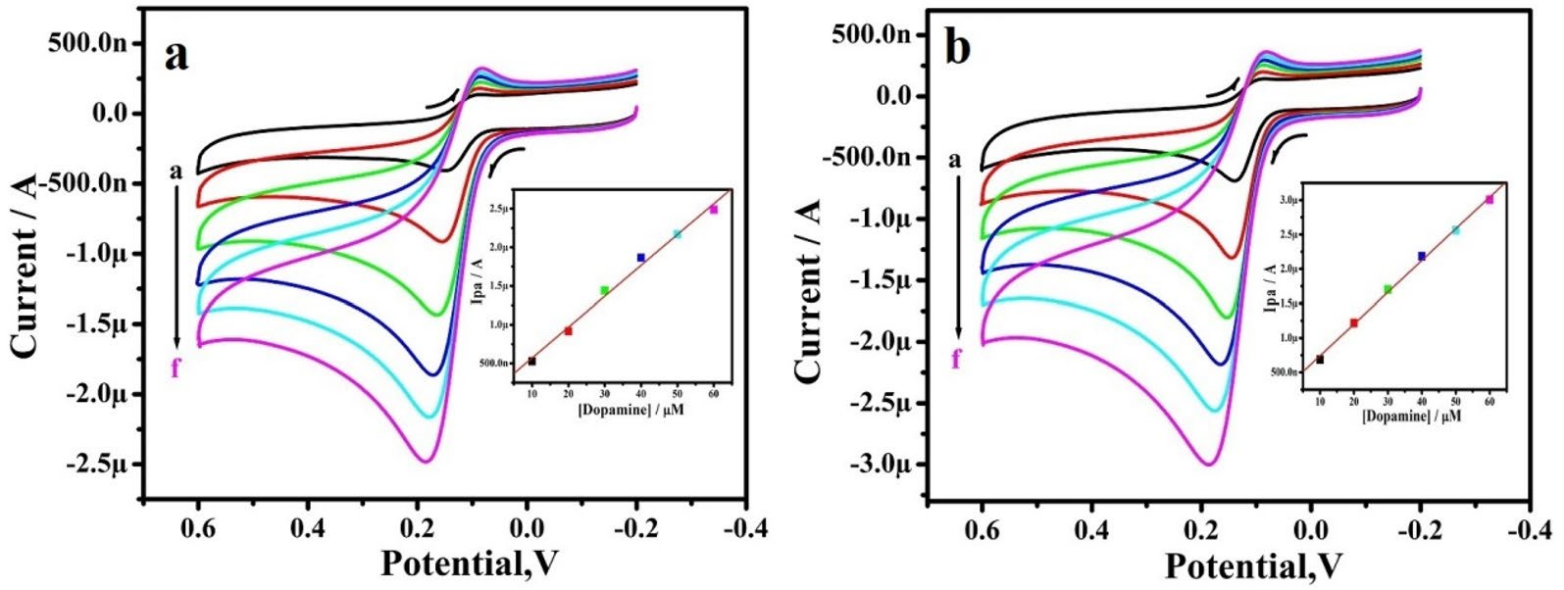
**(a)** CVs of DA (0.2 M PBS of pH 7.4) at RGO-Y_2_O_3_ MCPE with various concentrations. b**)** CVs of 10 μM DA (0.2 M PBS of pH 7.4) at RGO-Y_2_O_3_; Cr^3+^ MCPE with various concentrations. The inset figure **(a,b)** shows the graph of Ipa versus concentration of DA.

Figure 7a,b depicted the CVs of DA at RGO-Y_2_O_3_ and RGO-Y_2_O_3_Cr^3+^ MCPEs. The concentration of these biomolecules varied from 10 to 60 μM (a–f) in 0.2 M PBS at pH 7.4. The Ip of DA hikes with increase in the concentration. The plot of Ipa v/s DA concentration exhibits the correlation coefficient value was found to be 0.9954 and 0.9986 respectively (inset of Fig. 7a,b). The limit of detection (LOD) and limit of quantification (LOQ) was calculated by using the equations^27^:

3 $${\text{LOD }} = {\text{ 3 S}}/{\text{M}}$$

4 $${\text{LOQ }} = { 1}0{\text{ S}}/{\text{M}}$$

To determine, the LOD for DA was 6.01 and 3.26 µM. & LOQ was 20.04 and 10.88 µM for DA at the RGO-Y_2_O_3_ and RGO-Y_2_O_3_Cr^3+^ MCPEs respectively. The comparative analytical performance of electrode for DA is designed in Table 2^28–37^.

**Table 2:** Comparative analytical performance of electrode for DA.

Sl. no.	Electrode	Detection limit (µM)	Method	References
01	Au/Gr-Au	30	SW	^28^
02	Pt–Au hybrid	24	CV	^29^
03	CTAB/CPE	11.0	DPV	^30^
04	Fc-MCPE	9.4	CV	^31^
05	Poly (sudan III)/MCPE	9.3	CV	^32^
06	SWCNT/GCE	7.0	DPV	^33^
**07**	**RGO-Y**_**2**_**O**_**3**_** MCPE**	**6.01**	**CV**	**Present work**
08	Metallothioneins self-assembled gold electrode	6.0	CV	^34^
09	LDH/CILE	5.0	DPV	^35^
10	Ag-reduced GO/GCE	5.4	LSV	^36^
11	Ag/Ag2S-CNT-Nafion	4.7	DPV	^37^
**12**	**RGO-Y2O**_**3**_**Cr**^**3+**^** MCPE**	**3.26**	**CV**	**Present work**

### Influence of pH

**Figure 8:**
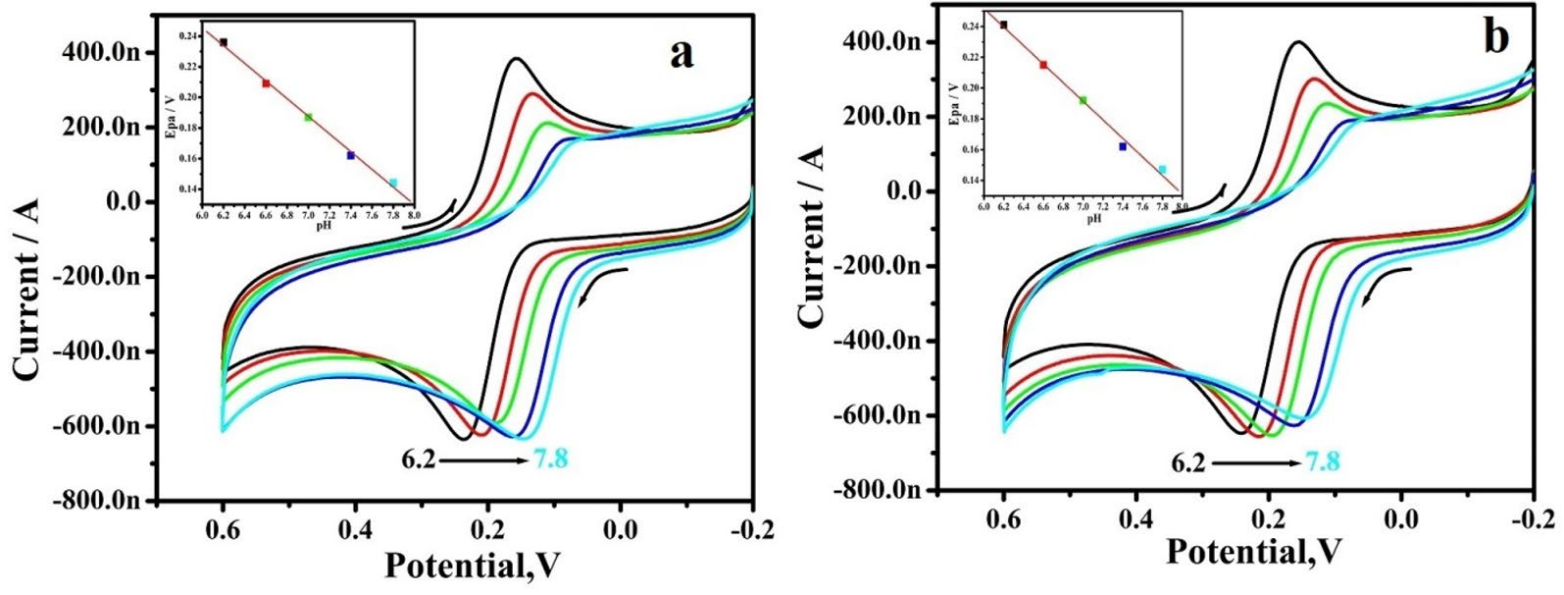
**(a)** CVs for different pH of 10 μM DA in 0.2 M PBS at RGO-Y_2_O_3_ MCPE, (**b)** CVs for different pH of 10 μM DA in 0.2 M PBS RGO-Y_2_O_3_; Cr^3+^ MCPE. The inset figure (**a**,**b)** shows the graph of Epa v/s pH.

The effect of pH on the electrochemical response of the dopamine at the RGO-Y_2_O_3_ and RGO-Y_2_O_3_Cr^+3^ MCPEs was carefully examined in the pH series of 6.2–7.8 shown in Fig. 8a,b respectively. The peak potential shifts to a negative side with hike pH in the MCPEs. The anodic peak potential (Epa) versus pH graph (inset of Fig. 8a,b) clearly illustrates. The getting slopes of 57 mV/pH and 60 mV/pH are very close to the Nernstian value of 59 mV for an equal number of electron and proton transfer reactions.

### Selectivity& stability

**Figure 9:**
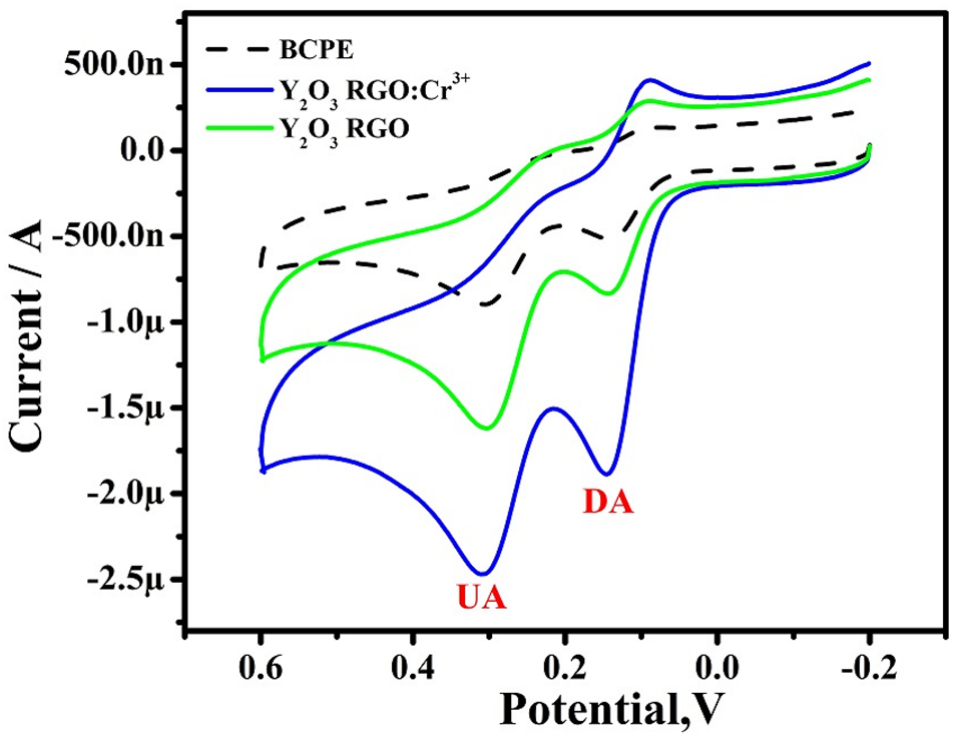
CVs for selectivity analysis for 10 µM DA & 10 µM UA at BCPE & MCPE at a scan rate of 50 mVs^-1^.

Selectivity & stability are also key indicators for practical use. The CVs were recorded for the mixture of 10 µM DA with 10 µM UA, in 0.2 M PBS of pH 7.4 at BCPE, RGO-Y_2_O_3,_ and RGO-Y_2_O_3_Cr^3+^ MCPEs (Fig. 9). The CVs responses for analytes with low current intensities were seriously overlapped, demonstrating the poor selectivity & sensitivity of the BCPE. However, in identical conditions, the MCPE can separate the oxidation potential of all analytes in the mixture. This result was identifying the selectivity of MCPE. The stability of RGO-Y_2_O_3_ and RGO-Y_2_O_3_Cr^3+^ was also investigated after being stored in a dry state for seven days at room temperature. It was found that the current signals retained 95.6% and 96.18% of the initial current response & the peak potentials were unchanged.

### Interference study

**Figure 10:**
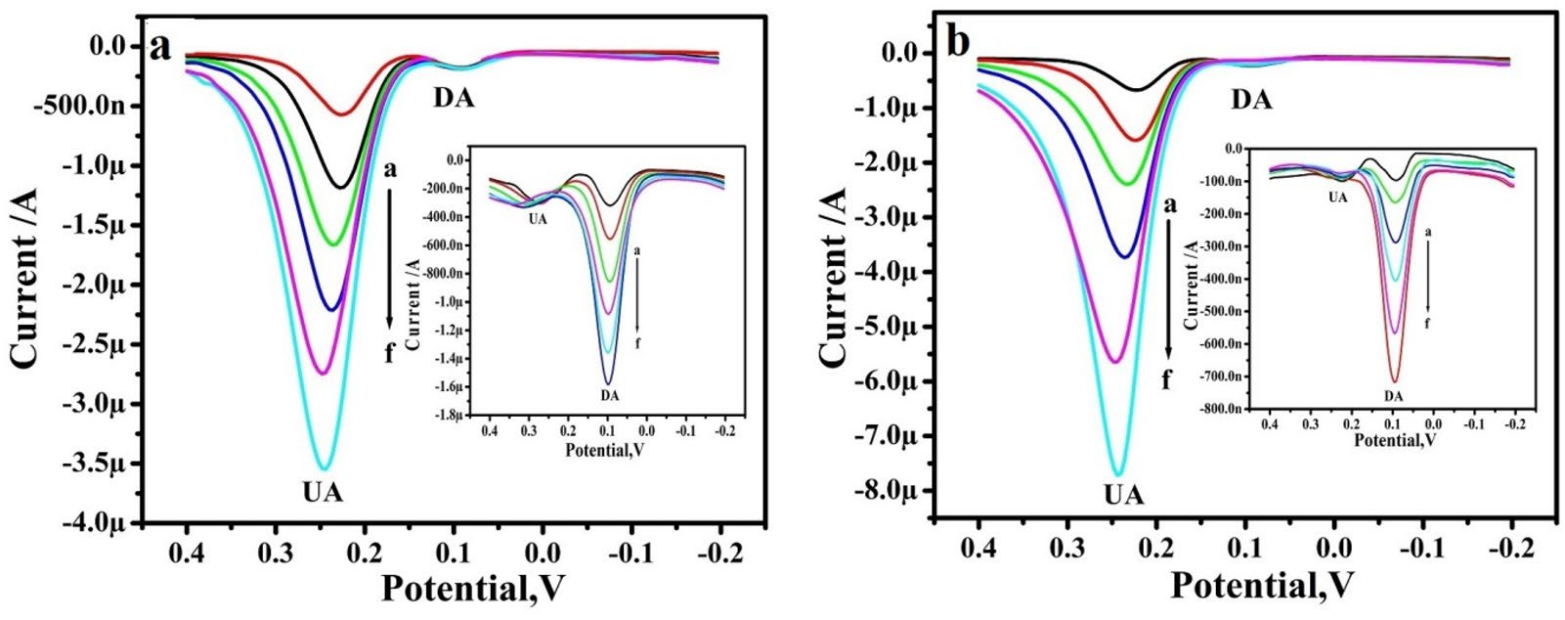
DPVs for different concentrations of 10–60 µM UA in PBS (pH 7.4) with 10 µM DA at (**a**) RGO-Y_2_O_3_ MCPE and (**b**) RGO-Y_2_O_3_; Cr^3+^ MCPE. The inset figure DPVs for different of concentrations of 10–60 µM DA in PBS (pH 7.4) with 10 µM UA at (inset **a)** RGO-Y_2_O_3_ MCPE and (inset **b**). RGO-Y_2_O_3_; Cr^3+^ MCPE.

Lastly, to evaluate the feasibility of the proposed method, the interference of possible chemicals in the determination of DA was conducted, the interference study was performed in the mixture of samples of DA and UA at the RGO-Y_2_O_3,_ and RGO-Y_2_O_3_Cr^3+^ MCPEs by differential pulse voltammetry (DPV). The RGO-Y_2_O_3_ MCPE (Fig. 10a) shows the Ipa of UA was increased with increased concentration from 10 to 60 µM by keeping the constant concentration of 10 µM DA, Similarly, DA concentration was varied and its Ipa is increased with the concentration (Inset Fig. 10a). The same procedure also adopted for RGO-Y_2_O_3_Cr^3+^ MCPE and varying the concentration of UA and DA shows in Fig. 10b (Inset Fig. 10b) respectively. This result shows higher current sensitivity and absence of background current and this result helps in the accurate and precise determination of DA and UA at RGO-Y_2_O_3_ and RGO-Y_2_O_3_Cr^3+^ MCPEs.

### The analytical application of the proposed sensor

To verify the success of the proposed sensor in the dopamine hydrochloride injection. The injection was procured from VHB Medi Sciences Ltd with a specified content of DA 40.0 mg/mL (suitable dilution in 0.2 M PBS). The samples were analyzed by the standard addition method. The results have been shown in Table 3. Therefore, the proposed modified electrode could be applied for the real sample analysis with satisfactory results.

**Table 3:** Detection of DA in the real sample (n = 2).

Electrodes	Sample added (µM)	Found (µM)	Recovery (%)
RGO-Y_2_O_3_ MCPE	20	19.48	97.4
	30	29.92	99.6
RGO-Y2O_3_Cr^3+^ MCPE	20	19.5	97.8
	30	29.96	99.8

## Conclusion

In the present work, the RGO-Y_2_O_3_ and RGO-Y_2_O_3_: Cr^+3^ NCs were synthesized and used as sensors for DA. The prepared RGO-Y_2_O_3_ and RGO-Y_2_O_3_: Cr^+3^ MCPEs shows good current response towards DA. We successfully studied the selectivity and stability of RGO-Y_2_O_3_ and RGO-Y_2_O_3_: Cr^+3^ MCPEs with the influence of pH. The RGO-Y_2_O_3_: Cr^+3^ MCPE shows good linearity with the lower LOD compared to other electrodes. Thus, the facile green synthesized RGO-Y_2_O_3_: Cr^+3^ NCs are a promising electrode material for sensor applications.
